# P-305. Empirical Treatment with vancomycin-resistant enterococcus (VRE) Effective Antibiotics versus Glycopeptides: Impact on Survival Outcomes in VRE Bloodstream Infections

**DOI:** 10.1093/ofid/ofae631.508

**Published:** 2025-01-29

**Authors:** Tao Hung Ou, Yu-Chung Chuang, Jann-Tay Wang, Yee-Chun Chen, Shan-Chwen Chang

**Affiliations:** National Taiwan University Hospital, Taipei, Taipei, Taiwan (Republic of China); National Taiwan University Hospital and National Taiwan University College of Medicine, Taipei, Taipei, Taiwan; National Taiwan University Hospital, Taipei, Taipei, Taiwan (Republic of China); National Taiwan University Hospital, Taipei, Taipei, Taiwan (Republic of China); National Taiwan University Hospital, Taipei, Taipei, Taiwan (Republic of China)

## Abstract

**Background:**

Vancomycin-Resistant Enterococcus (VRE) is a predominant pathogen in nosocomial bacteremia (BSI) in Taiwan. Although glycopeptides are traditionally recommended for empiric treatment of suspected Gram-positive infections, this study evaluates whether empiric anti-VRE antibiotics improves survival compared to glycopeptides following definitive VRE treatment confirmed by blood culture.

**Methods:**

This retrospective cohort study was conducted at the National Taiwan University Hospital and its branches from January 2010 to July 2023. It included patients aged 18 or older diagnosed with VRE BSI who received definitive treatment with linezolid or daptomycin (defined anti-VRE antibiotics). Patients were categorized into two groups based on the initial treatment received on the onset day of VRE BSI: empiric anti-VRE and empiric glycopeptide. The primary outcome was 28-day mortality.

**Results:**

Of 2,067 VRE BSI episodes recorded, 138 met the inclusion criteria. The average age was 64.1 years, with a male majority of 64.5%. The overall mortality rate was 55.1%. The empiric anti-VRE group included 50 patients, while the empiric glycopeptide group comprised 88 patients. Time to definitive treatment was significantly shorter for the empiric anti-VRE group (0 days) compared to the empiric glycopeptide group (2.2 days; P < 0.001). Mortality was lower in the empiric anti-VRE group at 42% versus 62.5% in the empiric glycopeptide group (P = 0.02). Multivariable logistic regression showed empiric anti-VRE treatment as a significant predictor of lower mortality (adjusted odds ratio [aOR] = 0.38, 95% CI: 0.17–0.85; P < 0.05), independent of the Charlson comorbidity index (aOR = 1.23; P = 0.008), steroid use (aOR = 2.61; P = 0.03), and the Pitt bacteremia score (aOR = 1.43; P < 0.001).

**Conclusion:**

Among patients who received definitive treatment for VRE, those treated with empiric anti-VRE demonstrated superior survival outcomes compared to those given empiric glycopeptides. This study underscores the importance of timely and appropriate antimicrobial therapy in managing VRE infections. While our data do not advocate for the universal use of empiric anti-VRE treatment, they suggest that such therapy should be considered when VRE infection is highly suspected to potentially improve outcomes.

**Disclosures:**

**All Authors**: No reported disclosures

